# Hippocampal neuroprotection mediated by secretome of human mesenchymal stem cells against experimental stroke

**DOI:** 10.1111/cns.13886

**Published:** 2022-06-18

**Authors:** Afsaneh Asgari Taei, Leila Dargahi, Pariya Khodabakhsh, Mehdi Kadivar, Maryam Farahmandfar

**Affiliations:** ^1^ Neuroscience Research Center Shahid Beheshti University of Medical Sciences Tehran Iran; ^2^ Department of Neuroscience and Addiction Studies, School of Advanced Technologies in Medicine Tehran University of Medical Sciences Tehran Iran; ^3^ Neurobiology Research Center Shahid Beheshti University of Medical Sciences Tehran Iran; ^4^ Department of Biochemistry Pasteur Institute of Iran Tehran Iran

**Keywords:** apoptosis, conditioned medium, inflammation, ischemic stroke, mesenchymal stem cells, neurogenesis, trophic factors

## Abstract

**Aims:**

Regenerative medicine literature has demonstrated that the therapeutic potentials of mesenchymal stem cells (MSCs) in experimental stroke are attributed to secreted bioactive factors rather than to cell replacement. Here, we explored the effects of secretome or conditioned medium (CM) derived from human embryonic stem cell‐derived MSCs (hESC‐MSCs) on hippocampal neurogenesis, inflammation, and apoptosis in experimental stroke.

**Methods:**

Ischemic stroke was induced by right middle cerebral artery occlusion (MCAO) in male Wistar rats, and CM was infused either one time (1‐h post‐stroke; CM1) or three times (1‐, 24‐, and 48‐h post‐stroke; CM3) into left lateral ventricle. Neurogenesis markers (Nestin, Ki67, Doublecortin, and Reelin) were assessed at transcript and protein levels in the dentate gyrus of the hippocampus on day seven following MCAO. In parallel, changes in the gene expression of markers of apoptosis (Bax and Bim, as well as an anti‐apoptotic marker of Bcl2), inflammation (IL‐1β and IL‐6, as well as IL‐10 as an anti‐inflammatory cytokine), trophic factors (BDNF, GDNF, NGF, and NT‐3), and angiogenesis (CD31 and VEGF) in the hippocampus were assessed.

**Results:**

Our results demonstrate that CM3 treatment could stimulate neurogenesis and angiogenesis concomitant with inhibition of inflammation, apoptosis, and neuronal loss in ischemic brains. Furthermore, rats treated with CM3 exhibited upregulation in neurotrophic factors.

**Conclusion:**

Our results suggest that hESC‐MSC‐CM could promote neurogenesis and protect brain tissue from ischemic injury, partly mediated by induction of angiogenesis and neurotrophic factors and inhibition of inflammatory and apoptotic factors expression.

## Graphical abstract

The secretome derived from human embryonic mesenchymal stem cells could promote hippocampal neurogenesis concomitant with inhibition of inflammation and apoptosis in ischemic brains.

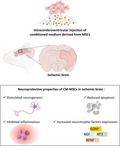

## 1 INTRODUCTION

Globally, ischemic stroke is one of the leading causes of death and long‐term disabilities.^1^ Ischemic stroke results from thrombotic or embolic occlusion of a major cerebral artery, leading to severe reduction or cessation of cerebral blood flow, and estimated that 85% of all strokes are ischemic.^2^ Cerebral ischemia/reperfusion involves many complex pathological processes including excitotoxicity, oxidative stress, blood–brain barrier (BBB) disruption, mitochondrial dysfunction, inflammation, and apoptosis, leading to neural cell death and impaired sensory, motor, and cognitive functions.^3^ Although intravenous thrombolysis and endovascular mechanical thrombectomy, which are widely used in treating ischemic stroke patients, aim to restore blood flow to the affected brain their applications are remained restricted by limited time window and related complications such as edema, intracranial hemorrhage, and hemorrhagic transformation.^4^, ^5^

Neurogenesis in neurogenic niches of the subventricular zone (SVZ) of the lateral ventricles and the subgranular zone (SGZ) of the hippocampal dentate gyrus (DG) is a compensatory adaptive mechanism to replacing lost neurons in the ischemic brain. Based on reports and observations, neurogenesis is considered an essential process in endogenous recovery following stroke. It involves the proliferation of neural stem/progenitor cells (NSPCs) to produce neuroblasts, migration of immature neuroblasts to the injured sites, and differentiation to functional neurons.^6^, ^7^ However, most newly formed cells undergo apoptosis, reflecting the undesirable microenvironment for survival, lack of trophic factors support and connections, and chronic inflammatory responses.^8^, ^9^

Currently, novel regenerative strategies such as stem cell‐based treatment focus on regenerative potentials of the damaged brain tissue.^7^ Among them, mesenchymal stem cells (MSCs) have quickly attracted scientific interest due to their tremendous capacity to be exploited in basic research and clinical settings. Many reports have demonstrated that the MSCs through different cellular signaling pathways stimulate critical processes involved in endogenous neuro‐restorative mechanisms in response to ischemic injury.^10^, ^11^ Although initially the therapeutic effects of transplanted MSCs mainly attributed to direct replacement of damaged cells in the ischemic brain, potential tumorigenicity, immunogenicity, unwanted homing into other organs, and low survival rate of transplanted MSCs are highlighting the significance of the paracrine activities or bystander effects of MSCs.^12^, ^13^ MSCs provide a proper milieu for tissue repair and regeneration through the secretion of diverse bioactive molecules, including trophic factors, growth factors, immunomodulatory cytokines, chemokines, and extracellular vesicles (EVs), overall known as the secretome.^13^, ^14^ Hence, cell‐free therapeutics utilization such as conditioned medium (CM) bypasses the current restrictions of cell therapy and exerts remarkable advantages over MSCs transplantation in stroke treatment.^15^, ^16^

Human embryonic stem cell‐derived MSCs (hESC‐MSCs) based on their high proliferative capacity, great anti‐inflammatory, and immunomodulatory properties are ideal sources in regenerative medicine.^17^, ^18^ We recently found that CM derived from hESC‐MSC (hESC‐MSC‐CM) could improve neurological deficits and reduce infarction volume in a rat model of focal cerebral ischemia.^19^, ^20^ Furthermore, treatment with CM promotes neurogenesis in SVZ neurogenic niche as well as ectopically in infracted regions.^20^ Therefore, we aimed to answer whether neurogenic effects of hESC‐MSC‐CM can also extend to the hippocampus of the ischemic brain. Besides, the expression levels of inflammatory markers, angiogenesis markers, apoptosis markers, and neurotrophic factors in response to ischemia insult were evaluated to address the presumptive other benefits of CM treatment on the hippocampus of ischemic brain.

## 2 MATERIALS AND METHODS

### 2.1 hESC‐MSCs conditioned medium preparation

Human ESC‐MSCs was initially characterized by evaluating their surface marker profiles and multi‐lineage differentiation capacity, as we described previously.^20^ Cells were cultured in low‐glucose Dulbecco's Modified Eagle's Medium (DMEM) (Gibco) supplemented with 10% fetal bovine serum (FBS), 2 mM L‐glutamine, 1% antibiotic/antimycotic (Gibco) at 37°C and 5% CO_2_. On reaching 80% confluency, cells were washed twice in phosphate buffer saline (PBS) to remove the serum and incubated for 24 h with a serum‐free DMEM containing 0.05% human serum albumin and 2 mM L‐glutamine. Then, cell‐free supernatants were aspirated and centrifuged (1000 *g*, 4°C, 15 min) to remove the cellular debris. The media was concentrated approximately 100‐fold by centrifugal ultrafilter membrane (3 kDa cut‐off, Millipore) and immediately kept at −80°C until further use.^21^ To minimize CM content variations and get the optimal consistency between experimental groups, hESC‐MSC‐CM required for all animals was gathered as a whole in batch and stored single‐use *aliquots*.

### 2.2 Animals

Adult male Wistar rats (260–290 g) were maintained under controlled temperature with a 12‐h light/12‐h dark cycle; and ad libitum access to food and water. All efforts were conducted to minimize the number of rats used and their suffering. All animal experiments were performed in accordance with the international guidelines for the Care and Use of Laboratory Animals (NIH Publications No. 80–23, revised, 1996), and research was approved by the Ethics Committee of Tehran University of Medical Sciences (IR.TUMS.REC.1394.1901). Furthermore, the animal data were reported in compliance with the ARRIVE guidelines 2.0 (ARRIVE, Animal Research: Reporting in Vivo Experiments).^22^

### 2.3 Stereotaxic surgery

Animals were anesthetized by ketamine and xylazine (65 and 15 mg/kg, respectively) and subjected to stereotaxic (Stoelting Instruments) surgery for guide cannula (22‐gauge) implantation into the left cerebral ventricle based on the Paxinos and Watson atlas with coordinates relative to the bregma (AP: −0.8 mm; ML: −1.5 mm; DV: 4 mm). Intracerebroventricular (ICV) correct cannulation was proved by evaluating the location of the cannula placement in brain slices of three rats.

### 2.4 MCAO procedure and experimental groups

To induce ischemic stroke, cannulated rats were initially anesthetized by isoflurane inhalation (5% induction and 2% maintenance) via an anesthesia machine (Parkland Scientific). The intraluminal filament model of middle cerebral artery occlusion (MCAO) was achieved through inserting silicone‐coated monofilament (Doccol 403556PK) via the right common carotid artery.^23^ Reperfusion of ischemic areas was induced by gently withdrawing the monofilament at 90‐min post‐occlusion's MCA. To prevent infection and dehydration post‐operation, enrofloxacin (5 mg/kg) and physiological saline (4 ml) were administrated subcutaneously daily for 3 days following MCAO. The body temperature of animals was maintained at 37°C using a heating pad.

**FIGURE 1:**
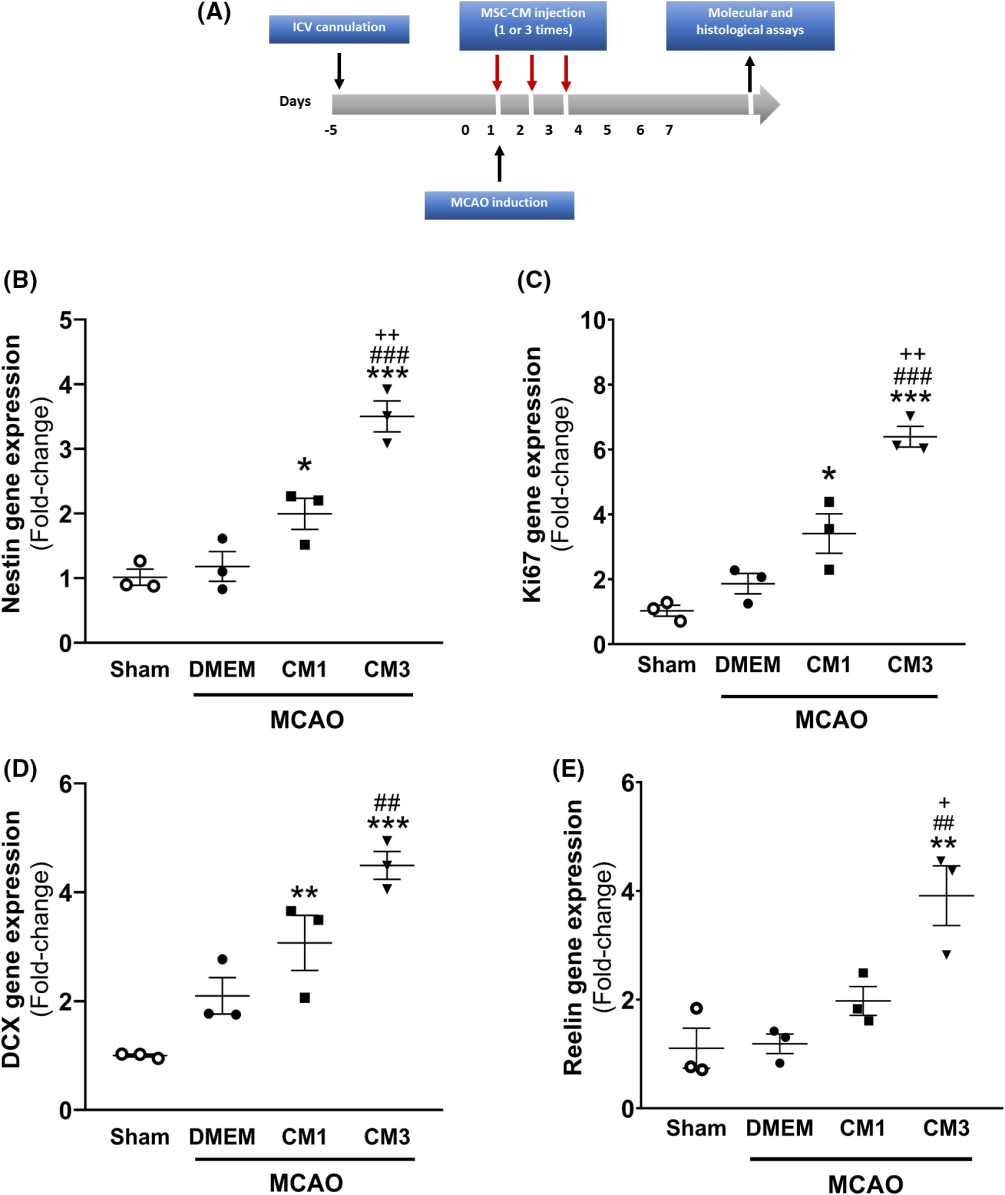
Effect of hESC‐MSC‐CM on mRNA levels of neurogenesis markers. (A) Schematic diagram of the experimental procedure. qPCR data analysis of (B) Nestin, (C) Ki67, (D) DCX, and (E) Reelin in the hippocampus. Data are reported as the mean ± SEM (*n* = 3). The differences between groups were determined by ANOVA followed by Tukey test. *p < 0.05, **p < 0.01 and ***p < 0.001 vs. Sham, ^##^ *p* < 0.01 and ^###^ *p* < 0.001 vs. MCAO+DMEM, ^+^ *p* < 0.05 and ^++^ *p* < 0.01 vs. CM1. CM, conditioned medium; DCX, Doublecortin; DMEM, Dulbecco's modified eagle's medium; MCAO, middle cerebral artery occlusion

Animals were randomly allocated to four groups: 1) Sham group: rats underwent stereotaxic surgery and the same MCAO manipulation without monofilament insertion. 2) Control group: rats received DMEM (1‐, 24‐, and 48‐h post‐MCAO). 3) MCAO+CM1 group: rats received CM (1‐h post‐MCAO). 4) MCAO+CM3 group: rats received CM (1‐, 24‐, and 48‐h post‐MCAO). The volume of DMEM and CM injected was 5 μl with 0.5 μl/min flow rate delivered via a 27‐gauge needle connected to a Hamilton syringe (Hamilton). The needle remained in place for 20 min before retraction to prevent any backflow up to the needle tract. A schematic diagram of the experimental procedure has been shown in Figure 1A.

### 2.5 RNA extraction and qPCR

On day 7 following MCAO, rats (*n* = 3) were sacrificed under deep anesthesia with CO_2_, hippocampus ipsilateral to ischemic areas immediately were dissected on ice, snap‐frozen, and subjected to the evaluation of transcripts analysis. Total RNA isolation from frozen tissues (Yekta Tajhiz Azma, Tehran, Iran, #YT9063) and subsequently reverse transcription of the extracted RNA into cDNA (Yekta Tajhiz Azma, Tehran, Iran, #YT4500) were carried out based on the manufacturer's protocols. The resulting sample cDNA was used to quantify target genes expression in five categories as follows 1) neurogenesis markers including Nestin, Ki67, Doublecortin (DCX), and Reelin; 2) factors involved in apoptosis including Bcl2, Bim, and Bax; 3) factors involved in inflammation including interleukin‐1β (IL‐1β), IL‐6, and Il‐10; 4) neurotrophic factors including brain‐derived neurotrophic factor (BDNF), glial cell‐derived neurotrophic factor (GDNF), nerve growth factor (NGF), and neurotrophin‐3 (NT‐3); 5) angiogenesis markers including CD31 and vascular endothelial growth factor (VEGF). To evaluate mRNA levels of target genes, qPCR was conducted on the ABI StepOne instrument (Thermo Scientific) with first‐strand cDNA, specific primers (presented in Table 1), and SYBR Green Master Mix reagent (RealQ Plus 2X, Ampliqon). The amplification conditions consisted of 5 min DNA polymerase activation at 95°C, 30 s denaturation at 95°C, 30 s annealing at optimum temperature, and 30 s extension at 72°C. The expression levels of target genes were normalized to HPRT1 level as an appropriate housekeeping gene in the experimental model of MCAO,^24^ and fold changes in cDNA levels were calculated by the 2^−ΔΔCt^ method.^25^, ^26^

**TABLE 1:** Primer (5′–3′) sequences used in qPCR

Gene	Forward	Reverse
Nestin	GGAGCAGGAGAAGCAAGGTC	GAGTTCTCAGCCTCCAGCAG
Ki67	CGGCGAGCCTCAAGAGATA	CGTGCTGTTCTACATGCCC
Doublecortin	GGAAGGGGAAAGCTATGTCTG	TTGCTGCTAGCCAAGGACTG
Reelin	GTCGTCCTAGTAAGCACTCGC	ACCTTCGCCTTCGGTTGTAG
IL‐1β	ACCCAAGCACCTTCTTTTCCTTC	GTCGTTGCTTGTCTCTCCTTGTA
IL‐6	GTATGAACAGCGATGATGCACTG	CATTGGAAGTTGGGGTAGGAAGG
IL‐10	GAAGCTGAAGACCCTCTGGATAC	CTCATTCATGGCCTTGTAGACACC
Bax	TGGTTGCCCTCTTCTACTTTGC	AAGTCCAGTGTCCAGCCCATG
Bim	ACAGAATCGCAAGACAGGAG	AGATAATGGTTGAAGGCCTGG
Bcl2	AGCCGGGAGAACAGGGTATG	TCTTCATCTCCAGTATCCCAC
BDNF	CAGAACAGAACAGAACAGAACAGG	CGATTAGGTGGCTTCATAGGAGAC
GDNF	CCTCTGCGACCTTTCCCTCTG	GCTGACCAGTGACTCCAATATGC
NGF	GAACAACATGGACATTACGCTATGC	CCCAATAAAGGCTTTGCCAAGGAC
NT‐3	ACTCTCCTCGGTGACTCTTATGC	GACACAGAACTACTACGGCAACAG
CD31	TGGAAGACCCGAGACTGAG	GAGGTATCGAATGGGCAGAA
VEGF	GCTCTCTTGGGTGCACTGGA	CACCGCCTTGGCTTGTCACA
HPRT	CCAGCGTCGTGATTAGTGATGATG	GAGCAAGTCTTTCAGTCCTGTCC

### 2.6 Tissue preparation and histological assays

To wash out the brain's blood and fixation of the cerebral parenchyma, anesthetized animals (*n* = 3) were perfused with ice‐cold PBS and 4% paraformaldehyde (PFA, Merck) transcardially on day 7 post‐MCAO. The harvested fixed brains were post‐fixed in PFA overnight, immersed in 30% sucrose solution (Merck) to cryoprotect the tissues, then covered with OCT compound, snap‐frozen, and stored at −80°C. Frozen sections of the hippocampus (−3.2 to −4.2 mm posterior to bregma) coronally sectioned at a thickness of 10 μm using a cryostat‐microtome (Sci Lab, English).

To immunofluorescence (IF) staining**,** hippocampal sections were subjected to antigen retrieval in sodium citrate buffer at 80°C for 30 min and permeabilized fixed cells with 1% Triton X‐100 in PBS (Merck). Following the blocking step with 10% normal goat serum diluted in PBST for 1 h at room temperature, overnight incubation of slides with primary specific antibodies including rabbit anti‐Nestin (1:100, Abcam, #ab93157), rabbit anti‐Ki67 (1:100, Abcam, #ab66155), and rabbit anti‐DCX antibodies (1:100, Abcam, #ab77450) was carried out at 4°C and subsequently brain sections for 1 h were treated with appropriate secondary antibody conjugated with FITC (1:50, Sigma, #F1262) at room temperature. Cell's nuclei were revealed with counterstaining DAPI, and result images at ×200 magnification were acquired using Nikon E600 fluorescent microscopy. The number of immunopositive cells in SGZ and hilus areas was assessed in at least three hippocampal sections per rat by ImageJ software. Data were normalized to the sham group and expressed as percentages relative to the sham group.^27^

For Nissl staining, following defatting and hydrating of hippocampal sections by xylene and descending graded ethanol, respectively, they were stained with 0.1% Cresyl Violet solution (Nissl Staining, Sigma‐Aldrich) for 6 min at 58°C. The sections were dehydrated, cleared, and cover‐slipped. Five stained sections from each animal were assessed by the light microscope (Nikon) at ×40 and ×200 magnifications. To evaluate neuronal survival in CA1, CA3, and DG subfields, round shape cells with a well‐defined nucleolus, and palely stained nuclei were considered as surviving neurons, while shrunken neurons with pyknotic and dark nuclei were considered as not surviving or dark neurons. The number of surviving neurons in the CA1, CA3, and DG subfields were counted in at least three brain sections (at ×200 magnification) per rat in different groups by ImageJ software and expressed as a percentage of the sham group.^28^

### 2.7 Statistical analysis

Values were represented as Mean ± SEM and analyzed by the 8th version of Graph Pad Prism (GraphPad Software). Shapiro–Wilk normality test indicated that the data have normal distribution; therefore, comparisons were done using parametric tests. Significance in molecular and histological evaluations was assessed by one‐way ANOVA followed by post hoc Tukey's test. *p* < 0.05 was considered for statistical significant.

## 3 RESULT

### 3.1 hESC‐MSC‐CM promoted neurogenesis in the hippocampus of MCAO rats

To evaluate neurogenesis, Nestin; as a NSPCs marker, Ki67; as a proliferation marker, and DCX; as migrating neuroblasts and immature neurons marker, have been widely used in different studies. Seven days following MCAO induction, the mRNA and protein levels of neurogenesis markers were investigated in the hippocampus by qPCR analysis and IF staining. In addition to classical neurogenesis markers, Reelin gene expression was assessed, which contributed to proper neuronal migration and synaptogenesis. One‐way ANOVA analysis indicated that MCAO was associated with the increased transcript number of neurogenesis markers compared with sham, although they were not statistically significant. Furthermore, CM3 treatment could significantly upregulate mRNA levels of Nestin (Figure 1B, *p* < 0.001), Ki67 (Figure 1C, *p* < 0.001), DCX (Figure 1D, *p* < 0.01), and Reelin (Figure 1E, *p* < 0.01) compared with MCAO.

**FIGURE 2:**
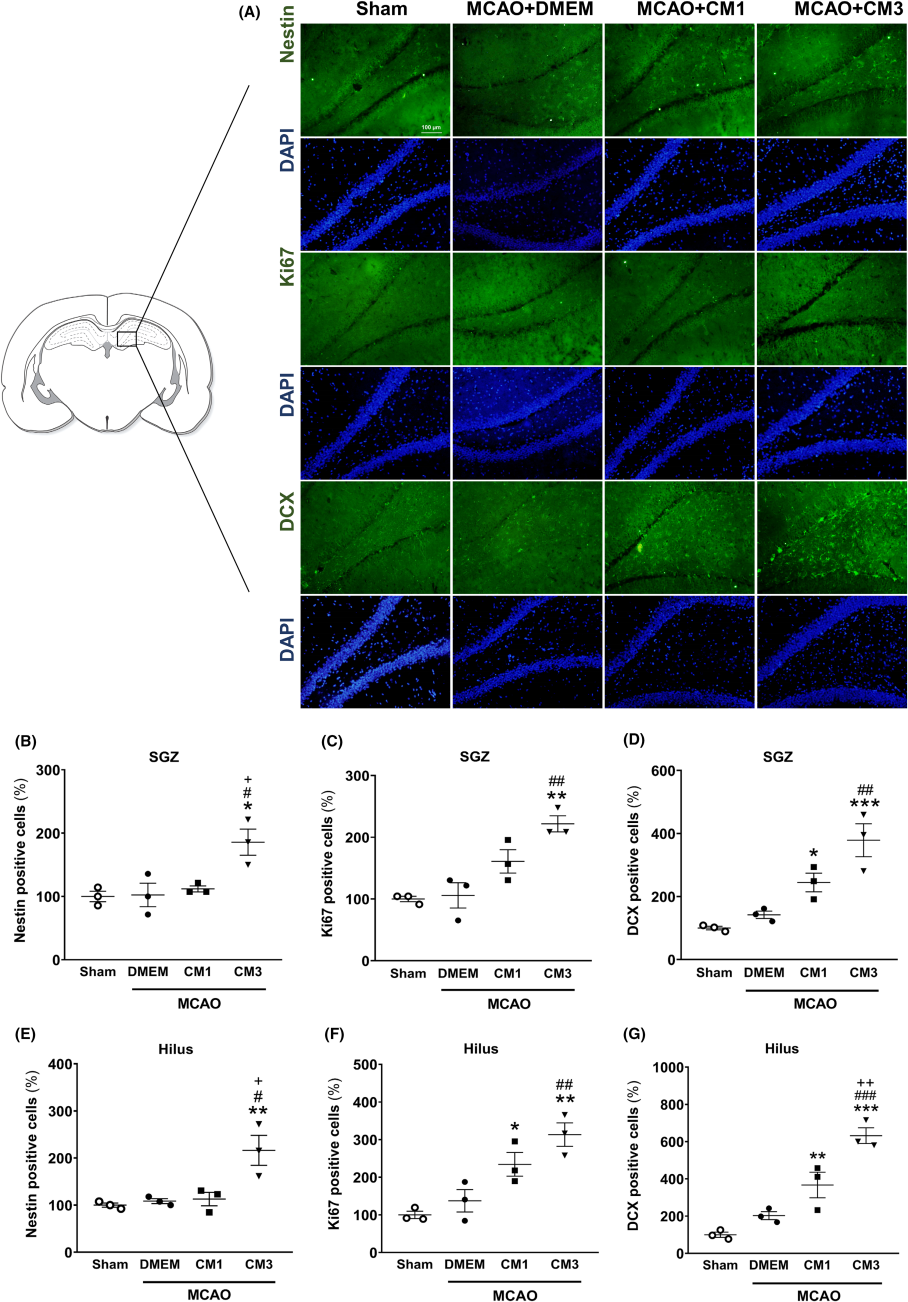
Effect of hESC‐MSC‐CM on protein expression of neurogenesis markers. (A) Representative micrographs of immunofluorescence staining of Nestin, Ki67, and DCX. Cell nuclei were counterstained with DAPI. Scale bar: 100 μm. The percentage of (B) Nestin, (C) Ki67, and (D) DCX‐ positive cells relative to sham in the SGZ. The percentage of (E) Nestin, (F) Ki67, and (G) DCX‐ positive cells relative to sham in the hilus. Data are reported as the mean ± SEM (*n* = 3); the differences between groups were determined by ANOVA followed by Tukey test. *p < 0.05, **p < 0.01, and ***p < 0.001 vs. Sham, ^#^ *p <* 0.05, ^##^ *p* < 0.01 and ^###^ *p* < 0.001 vs. MCAO+DMEM, ^+^ *p* < 0.05 and ^++^ *p* < 0.01 vs. CM1. CM, conditioned medium; DCX, Doublecortin; DMEM, Dulbecco's modified eagle's medium; MCAO, middle cerebral artery occlusion; SGZ, subgranular zone

As shown in Figure 2A, in IF staining, protein levels of Nestin, Ki67, and DCX‐positive cells were significantly increased in the SGZ neurogenic niche of rats receiving three injections of CM compared with the untreated stroke rats (Figure 2B–D, *p* < 0.05, *p* < 0.01, and *p* < 0.01, respectively). Furthermore, Nestin, Ki67, and DCX‐positive cells were also enhanced ectopically in the hilus of ischemic rats subjected to CM3 (Figure 2E–G, *p* < 0.05, *p* < 0.01, and *p* < 0.001, respectively).

### 3.2 hESC‐MSC‐CM modulated inflammation in the hippocampus of MCAO rats

**FIGURE 3:**
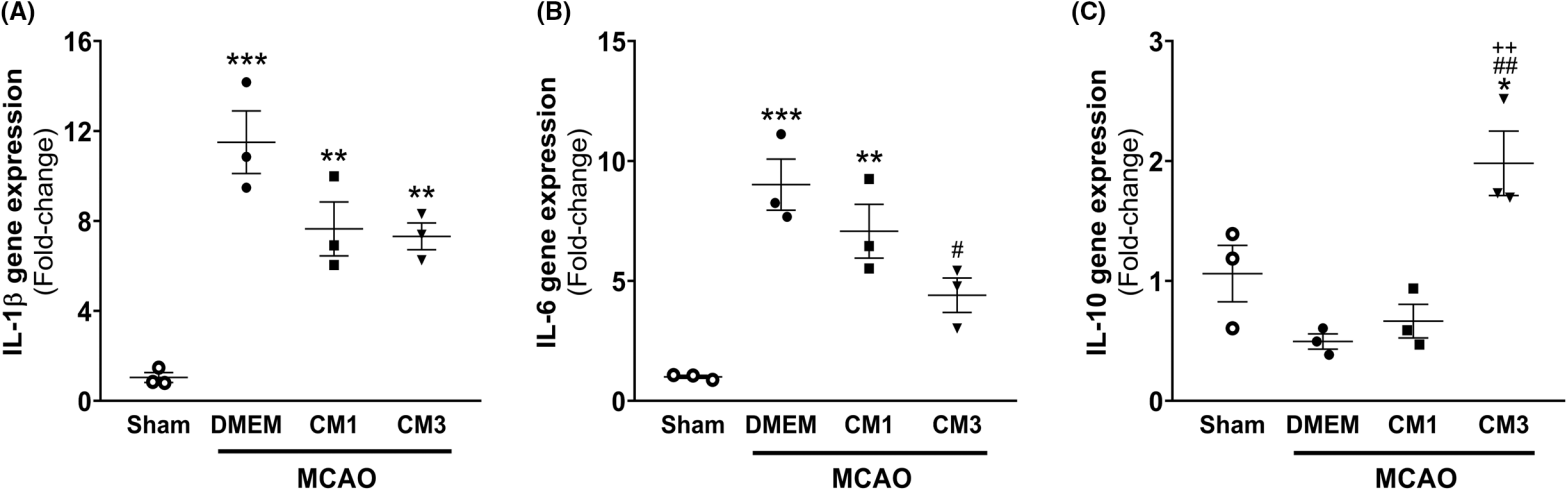
Effect of hESC‐MSC‐CM on mRNA levels of inflammatory markers. qPCR data analysis of (A) IL‐1β, (B) IL‐6, and (C) IL‐10 in the hippocampus. Data are reported as the mean ± SEM (*n* = 3). The differences between groups were determined by ANOVA followed by Tukey test. *p < 0.05, **p < 0.01, and ***p < 0.001 vs. Sham, ^#^ *p* < 0.05 and ^##^ *p* < 0.01 vs. MCAO+DMEM, ^++^ *p* < 0.01 vs. CM1. CM, conditioned medium; DMEM, Dulbecco's modified eagle's medium; IL, interleukin; MCAO, middle cerebral artery occlusion

To investigate ESC‐MSC‐CM effect on the inflammatory response, mRNA expression levels of IL‐1β and IL‐6 as pro‐inflammatory mediators and IL‐10 as an anti‐inflammatory cytokine in the hippocampus were assessed on day 7 post‐injury. The one‐way ANOVA analysis revealed that IL‐1β and IL‐6 transcripts were upregulated in response to stroke (*p* < 0.001), while mRNA expression of IL‐10 did not change relative to sham (*p* = 0.20). Furthermore, our results showed that rats treated with three injections of hESC‐MSC‐CM exhibited nonsignificant alteration in IL‐1β transcript (Figure 3A, *p* = 0.60) as well as a significant reduction in IL‐6 mRNA levels compared with the MCAO group (Figure 3B, *p* < 0.05). Treatment with CM3 could significantly upregulate the expression level of IL‐10 compared with control rats receiving DMEM (Figure 3C, *p* < 0.01).

### 3.3 hESC‐MSC‐CM reduced apoptosis in the hippocampus of MCAO rats

**FIGURE 4:**
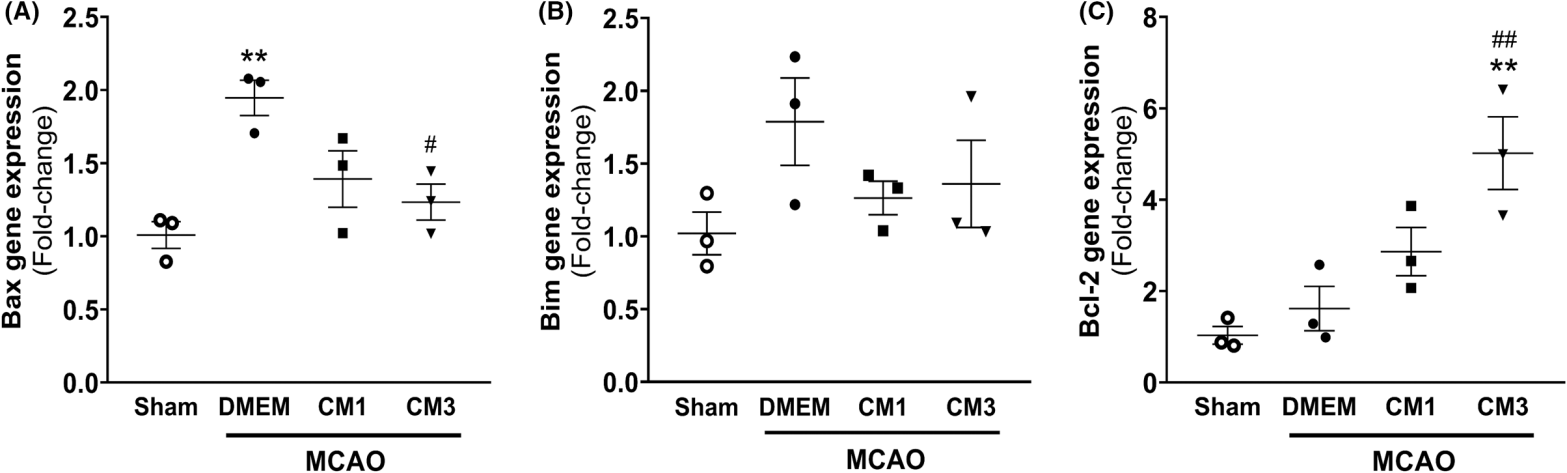
Effect of hESC‐MSC‐CM on mRNA levels of apoptotic markers. qPCR data analysis of (A) Bax, (B) Bim, and (C) Bcl2 in the hippocampus. Data are reported as the mean ± SEM (*n* = 3). The differences between groups were determined by ANOVA followed by Tukey test. **p < 0.01 vs. Sham, ^#^ *p* < 0.05 and ^*##*^ *p* < 0.01 vs. MCAO+DMEM. MCAO+DMEM, ^++^ *p* < 0.01 vs. CM1. CM, conditioned medium; DMEM, Dulbecco's modified eagle's medium; MCAO, middle cerebral artery occlusion

Seven days following MCAO, mRNA levels of Bax and Bim, as pro‐apoptosis markers as well as anti‐apoptotic marker of Bcl2 were evaluated by qPCR analysis to determine the ESC‐MSC‐CM impact on apoptotic cell death. As depicted in Figure 4, the mRNA level of Bax increased following the MCAO compared with the sham group (*p* < 0.01), although no significant alterations were found in Bim and Bcl2 transcripts in the hippocampus after MCAO (*p* = 0.16 and *p* = 0.86, respectively). In rats receiving three injections of ESC‐MSC‐CM, expression levels of Bax were significantly downregulated relative to the MCAO group (Figure 4A, *p* < 0.05). However, no significant alteration was observed in mRNA levels of Bim in MCAO rats subjected to CM treatment (Figure 4B, *p* > 0.05). Furthermore, rats treated to CM3 exhibited a significant upregulation in Bcl2 transcripts compared with the control group (Figure 4C, *p* < 0.01).

### 3.4 hESC‐MSC‐CM increased the expression of the neurotrophic factors in the hippocampus of MCAO rats

**FIGURE 5:**
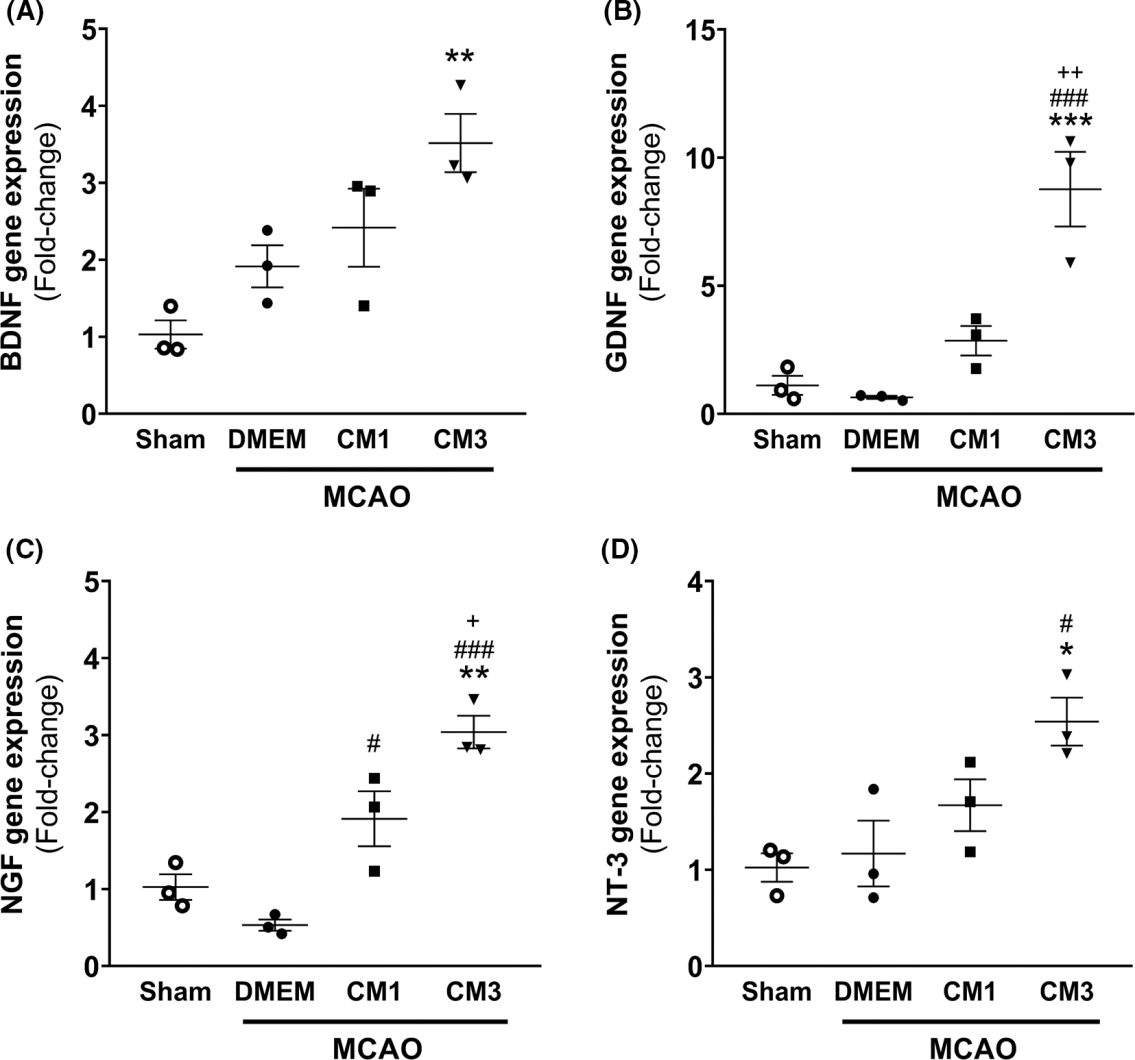
Effect of hESC‐MSC‐CM on mRNA levels of neurotrophic factors. qPCR data analysis of (A) BDNF, (B) GDNF, (C) NGF, and (D) NT‐3 in the hippocampus. Data are reported as the mean ± SEM (*n* = 3). The differences between groups were determined by ANOVA followed by Tukey test. *p < 0.05, **p < 0.01 and ***p < 0.001 vs. Sham, ^#^ *p* < 0.05 and ^###^ *p* < 0.001 vs. MCAO+DMEM, ^+^ *p* < 0.05 and ^++^ *p* < 0.01 vs. CM1. CM, conditioned medium; DMEM, Dulbecco's modified eagle's medium; MCAO, middle cerebral artery occlusion; BDNF, brain‐derived neurotrophic factor; GDNF, glial cell‐derived neurotrophic factor; NGF, nerve growth factor; NT‐3, neurotrophin‐3

The neurotrophic factors transcripts including BDNF, GDNF, NGF, and NT‐3 were measured 7‐day post‐stroke. The one‐way analysis indicated that relative expression of neurotrophic factors in the hippocampus region of the MCAO group was not altered compared with the sham rats. As represented in Figure 5, the mRNA level of BDNF increased in CM3 group compared with the sham group (Figure 5A, *p* < 0.01). Furthermore, three times ICV injections of ESC‐MSC‐CM could significantly upregulate mRNA levels of GDNF and NT‐3 relative to the control group (Figure 5B, *p* < 0.001 and Figure 5D, *p* < 0.05, respectively). Moreover, significant enhancement in NGF gene expression was observed in response to both CM1 and CM3 treatments compared with rats receiving DMEM (Figure 5C, *p* < 0.05 and *p* < 0.001, respectively).

### 3.5 hESC‐MSC‐CM stimulated angiogenesis in the hippocampus of MCAO rats

**FIGURE 6:**
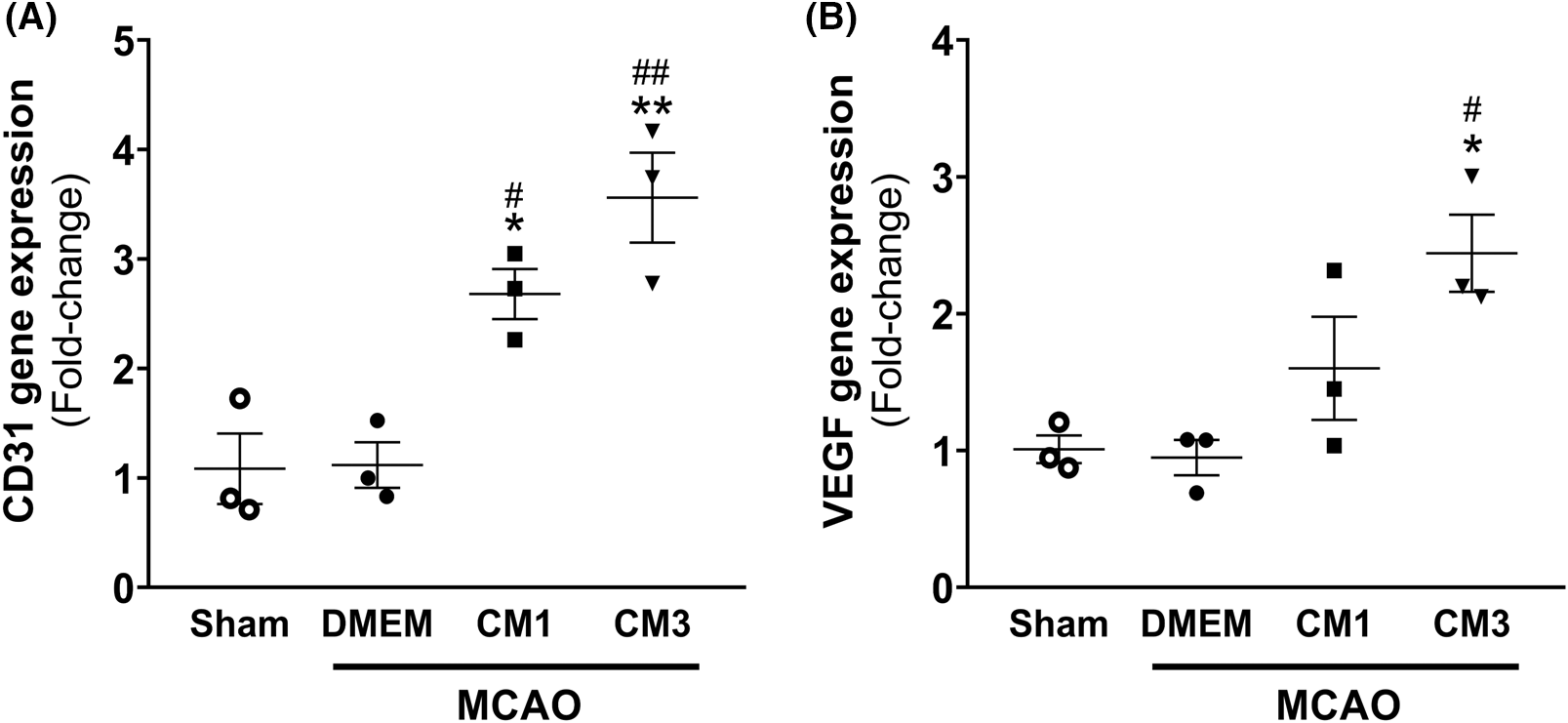
Effect of hESC‐MSC‐CM on mRNA levels of angiogenesis markers. qPCR data analysis of (A) CD31 and (B) VEGF in the hippocampus. Data are reported as the mean ± SEM (*n* = 3). The differences between groups were determined by ANOVA followed by Tukey test. *p < 0.05, **p < 0.01 and ***p < 0.001 vs. Sham, ^#^ *p* < 0.05 and ^###^ *p* < 0.001 vs. MCAO+DMEM, ^+^ *p* < 0.05 and ^++^ *p* < 0.01 vs. CM1. CM, conditioned medium; DMEM, Dulbecco's modified eagle's medium; MCAO, middle cerebral artery occlusion; VEGF, vascular endothelial growth factor

The transcript levels of CD31 and VEGF, as angiogenic markers, were assessed by qPCR analysis to evaluate the ESCs‐MSCs‐CM effect on angiogenesis. As represented in Figure 6A, the transcript level of CD31 significantly elevated following the treatment with CM1 and CM3 relative to MCAO rats (*p* < 0.05 and *p* < 0.01, respectively). Furthermore, mRNA level of VEGF was significantly increased in rats subjected to three injections of CM (Figure 6B, *p* < 0.05).

### 3.6 hESC‐MSC‐CM attenuated neurodegeneration in the hippocampus of MCAO rats

**FIGURE 7:**
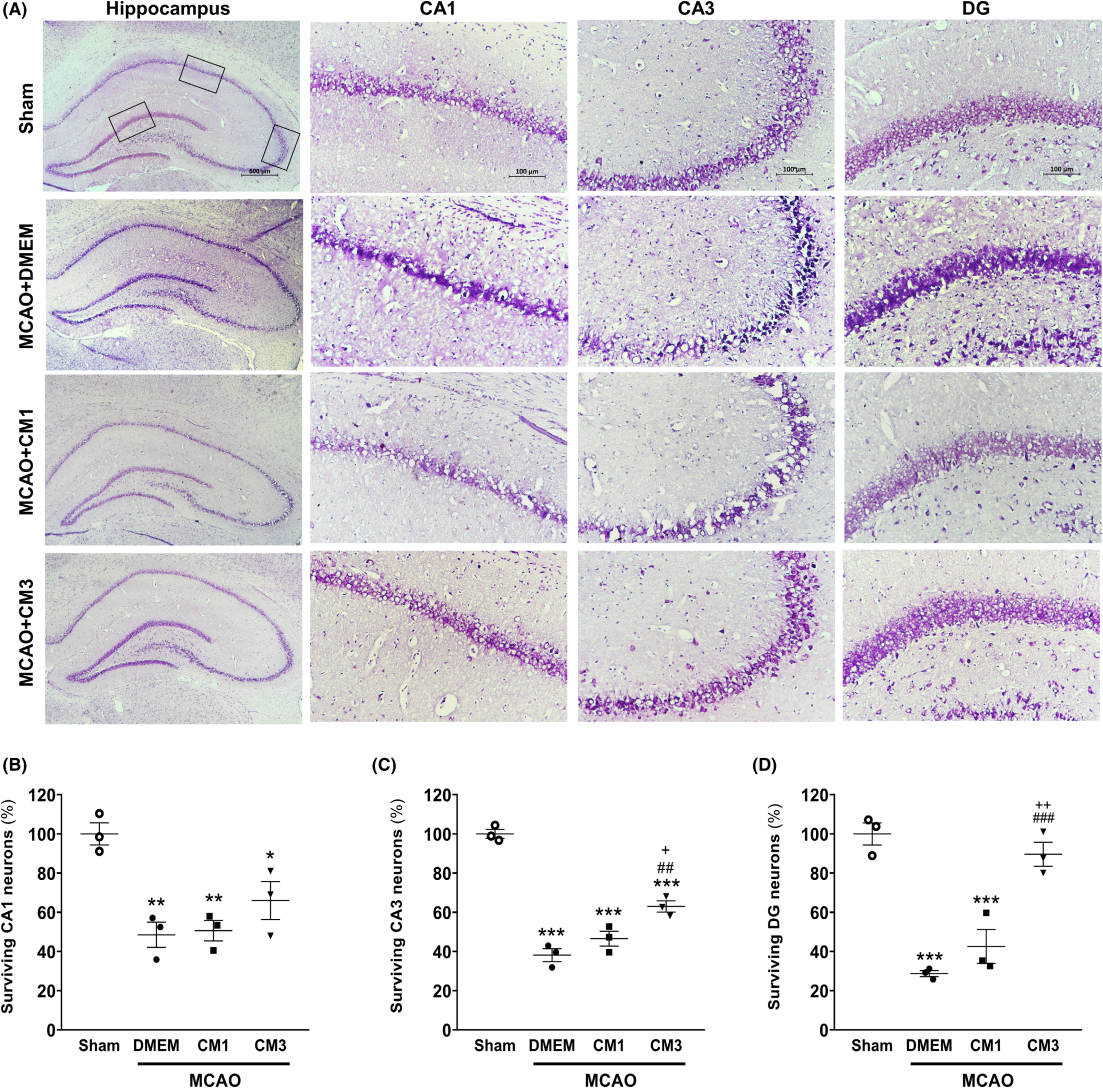
Effect of hESC‐MSC‐CM on neuronal survival. (A) Representative micrographs of Nissl‐stained sections in the CA1, CA3, and DG hippocampal subfields. Scale bar: 100 μm. (*n* = 3). The percentage of surviving neurons in (B) CA1, (C) CA2, and (D) DG in the hippocampus. *p < 0.05, **p < 0.01 and ***p < 0.001 vs. Sham, ^##^ *p* < 0.01 and ^###^ *p* < 0.001 vs. MCAO+DMEM, ^+^ *p* < 0.05 and ^++^ *p* < 0.01 vs. CM1. CM, conditioned medium; DMEM, Dulbecco's modified eagle's medium; MCAO, middle cerebral artery occlusion; DG, dentate gyrus

To evaluate neuronal degeneration post‐stroke, Nissl staining was carried out. As indicated in Figure 7A, neuronal loss was observed in the MCAO group in comparison with sham rats, and Nissl‐stained dark neurons with abnormal morphologies of massive shrunken were detected in a large number in all subfields. The one‐way ANOVA analysis showed that the percentage of surviving neurons in CA1, CA3, and DG was significantly reduced in the MCAO group in comparison with sham rats (Figure 7, *p* < 0.01, *p* < 0.001, and *p* < 0.001, respectively). Furthermore, CM3 treatment increased the number of surviving neurons in CA3 and DG regions of the hippocampus compared with ischemic rats (*p* < 0.01 and *p* < 0.001, respectively).

## 4 DISCUSSION

This study provides evidence that treating ischemic rats with hESC‐MSC‐CM exhibits protective effects against stroke insult through promoting neurogenesis and angiogenesis, inhibiting inflammation and apoptosis, and increasing neurotrophic factors expression. Therefore, MSCs‐derived products such as CM may hold great promise candidates in clinical protocols against ischemic stroke.

Although many studies suggest that MSCs derived from adult tissues such as bone marrow (BM)‐MSCs contribute to functional recovery in stroke models, some concerns including invasive harvesting methods, limited proliferation rate, senescence, and cellular heterogeneity lead to restriction of their utilizations in the clinical setting. hESC‐MSC, as an alternative younger origin, display some of the ESCs' unique features such as great proliferative potential as well as MSCs advantageous features such as the inability to teratoma development.^29^ As a renewable and homogenous source, these cells elevate high batch‐to‐batch consistency, reproducible efficacy, and, therefore, secretory factors.^30^ Moreover, great immunomodulatory capacity of hESC‐MSCs becomes them a promising and exciting option in cell‐based therapies.

Although the ICV injection, as an invasive route, has its own limitations, it is still considered as a valuable strategy to deliver high concentrations of therapeutics to the central compartment in patients with neurological disorders. A main advantage of ICV injection is that the trophic factors contained in CM can bypass the BBB to reach the brain via the cerebrospinal fluid. It also reduces the risk of adverse effects and the volume of CM required.^31^, ^32^

The preclinical stroke studies report neurological functions and infarction volume as primary endpoints, which these evaluations contribute to moving a drug into clinical research. We previously reported that ICV injection of hESC‐MSC‐CM could ameliorate neurological deficits, evidenced by decreased modified neurological severity scores, Bederson's scores, and the forelimb use asymmetry in MCAO rats. Moreover, ischemic rats subjected to CM treatment exhibited a reduction in infarction volumes.^19^, ^20^

Adult neurogenesis, as the intrinsic capability to self‐repair, is stimulated under certain pathological conditions such as ischemic insult. The stroke leads to activation of some process of endogenous self‐repair and neurogenic burst response in SVZ and SGZ.^33^, ^34^ Due to absence of trophic support and functional connections, most of newborn neurons fail to survive over long‐term.^6^, ^35^ Many studies highlighted the contribution of post‐stroke neurogenesis in functional recovery, and manipulations of stroke‐induced neurogenesis have been targeted as promising approaches in clinical settings for the treatment of neural disabilities following ischemic stroke.^8^, ^36^ Interestingly, preventing neurogenesis by targeted depletion of DCX‐expressing cells exacerbates neurological deficits and infarct size even on 1 day after MCAO.^37^ This effect occurs too short to be explained with the absence of new mature neurons generation, which needs weeks rather than days. So, it appears that the newly generated cells can enhance brain repair through not only neural replacement, but also trophic/homeostatic supports.^37^ We have previously revealed that treatment with hESC‐MSC‐CM could enhance NSCs pool, their proliferation, and neuroblast migration from ipsilateral SVZ toward injured striatum and cortex.^20^ Although MCAO induction has no significant effect on transcript and protein levels of neurogenesis markers in the present study, rats treated with three CM injections exhibited high Nestin, Ki67, and DCX in mRNA and protein levels on 7 day following stroke. In line with our findings, Tsai et al., reported that rats treated with intravenous injection of BM‐MSC‐CM exhibited enhancement of DCX‐positive cells in lateral ventricle near hippocampus.^38^ Intravenous infusion of CM from human normoxic‐ and hypoxic BM‐MSCs could also stimulate neurogenesis in peri‐lesioned cortex of rats on day four following traumatic brain injury.^39^

Interestingly, treatment with CM not only elevates NSPCs pool, their proliferation and differentiation in the SGZ neurogenic niche, but also potentiates ectopic neurogenesis in the hilus ischemic rat. Some new granule cells born with aberrant morphology have been observed located ectopically in the hilus ischemic brain. It seems that this aberrant neurogenesis may contribute to functional impairments, cognitive deficits, or epilepsy often seen in patients with stroke.^40^, ^41^ Although, Zhu et al. have reported that ablation of hilar ectopic neurogenesis fails to ameliorate subsequent cognitive deficits in epileptic mice.^42^

Consistently with classic neurogenesis markers, we also found increased mRNA level of Reelin in response to CM3 treatment, while did not affect Reelin gene expression in the MCAO group compared with sham. Reelin, as a serine protease of the extracellular matrix, is essential for neuronal layering and proper migration during development and in the adult nervous system.^43^ Furthermore, it is contributed to the maturation of dendritic spines, synaptogenesis, and neurites outgrowth in the hippocampus.^44^ Reelin is involved in neuronal survival and differentiation through activation of phosphoinositide 3‐kinases (PI3K) and mitogen‐activated protein kinase (MAPK) cascades.^45^ Reelin overexpression has been found to increase SGZ neurogenesis, synaptic contacts, dendritic spines hypertrophy, and long‐term potentiation responses in the adult hippocampus.^46^ In this regard, reelin‐deficient mice exhibited impaired neurogenesis in SGZ and deteriorated infarction volume post‐stroke.^47^

Angiogenesis is a multi‐step biological process for vascular network remodeling that is involved in restoring cerebral blood flow to ischemic areas and recovery from stroke.^6^ It is involved in cellular survival in the penumbra through upregulating the expressions of growth factors and neuroblasts migration to the ischemic region.^48^ Our findings showed that treatment with CM upregulates CD31 and VEGF expressions in MCAO rats. VEGF regulates endothelial cell proliferation, migration, sprouting activity, and the formation of immature vessels.^49^ Furthermore, VEGF exhibits anti‐apoptotic effects and promotes survival of migrating neuroblasts to the peri‐infarct area.^50^ In line with our findings, Cho et al. have shown that CM derived from human MSCs elevates number of CD31‐positive microvessels in the penumbra following MCAO.^16^ Recently, it has been reported that three times systemic injections of EVs derived from human BM‐MSCs could increase number of CD31^+^/BrdU^+^ microvessels and promote angiogenesis in the peri‐infarct cortex of ischemic rats.^51^ It should be noted the newly generated capillaries may also pose major concerns including edema formation, hemorrhagic transformation, and BBB damage. For instance, although VEGF is the most potent trigger for inducing angiogenesis, it also enhances vascular permeability. Thus, the nature of angiogenesis could be beneficial at appropriate time window, its pathophysiologic risk factors need to be critically considered.^52^

Neural cells in the surrounding penumbra region undergo apoptosis from hours to days following ischemia, suggesting that apoptosis, as a main cellular death pathway, represents a critical therapeutic target in stroke treatment.^53^ We showed that ischemic stroke accompanies apoptosis and MCAO rats subjected to CM3 exert downregulation of Bax and upregulation of Bcl2 in the hippocampus. In line with our findings, other studies also confirm anti‐apoptotic properties of MSC‐CM in acute and chronic phases following stroke.^54^, ^55^ Furthermore, CM from human amniotic fluid stem cells can protect SH‐SY5Y cells by activating prosurvival and anti‐apoptotic pathways against oxygen and glucose deprivation.^56^ The MSCs through upregulating anti‐apoptotic proteins and downregulating pro‐apoptotic proteins play a critical role in cellular survival.^15^, ^57^ In addition to directly inhibition of apoptosis, they mediate neuronal survival via releasing neurotrophic factors and their interaction with tyrosine kinase receptors and ultimately activation of PI3K/Akt pathway.^58^

Cerebral ischemia activates both innate and adaptive immune cells, which through producing pro‐inflammatory cytokines, chemokines, and reactive oxygen species leading to blood–brain barrier disruption and infiltration of a wide range of immune cells. These might contribute to amplify of the inflammatory cascades and further progression of tissue damage.^59^ In the present study, concomitant with apoptosis, we found a neuroinflammatory response manifested by the upregulation of IL‐1β and IL‐6 transcripts as pro‐inflammatory cytokines. Furthermore, we also showed that rats receiving three injections of hESC‐MSC‐CM exhibit decreased mRNA level of IL‐6 as well as increased mRNA level of IL‐10 relative to ischemic rats. Similarly, intravenous injection of EVs obtained from MSCs has been shown to induce anti‐inflammation properties and reduce infiltration of immune cells in ischemic brains of aged mice on 7‐day post‐MCAO as well as microglia accumulation in the peri‐infarct cortex of young rats on 28‐day post‐MCAO.^51^, ^60^ Moreover, in several studies strong anti‐inflammatory and immunomodulatory properties of ESC‐MSC have been revealed. They secrete higher anti‐inflammatory cytokines IL‐10 while releasing lower pro‐inflammatory cytokine IL‐6 than fetal MSCs or BM‐MSCs.^21^, ^61^, ^62^ In this regard, ESC‐MSCs increase nuclear factor kappa B (NF‐κB) signaling activation, leading to enhancement in downstream targets transcription, including anti‐inflammatory cytokines.^61^

There is a mutual interaction between inflammation and neurogenesis. It has been demonstrated that proliferation and migration of neural progenitor cells are affected by both pro‐ and anti‐inflammatory mediators, leading to different effects of inflammation on neurogenesis.^63^ As negative regulators, the pro‐inflammatory mediators, such as IL‐6, can interfere with adult hippocampal neurogenesis.^64^ In this study, increased pro‐inflammatory cytokines in MCAO rats inhibit hippocampal neurogenesis in the sub‐acute phase. Treatment with CM3 can promote proliferation and migration of NSPCs in DG ischemic rats partly through immunomodulation mechanisms. In line with our findings, the proliferation, survival, and differentiation of NSPCs in the SGZ have been shown to be downregulated via IL‐6 production.^64^ It has also been reported that the proliferation of progenitor cells was suppressed via TNF‐α secretion by activated microglia after stroke.^65^

In contrast to acute microglia activation, their chronic activation results in NSPCs survival and neuroprotective effects. These different responses may be attributed to the dual roles of microglia after stroke and microglia response has both beneficial and adverse outcomes for neurogenesis.^63^ On the contrary, NSPCs could promote neuroprotection via maintaining undifferentiated properties and immune‐like functions in a mouse model of chronic neuroinflammation.^66^

Here, we demonstrated that treatment with hESC‐MSC‐CM gave rise to neuroprotective effects against cerebral insult, which are manifested through neurogenesis induction as well as inhibition of apoptosis and inflammation. Gene expression of neurotrophic factors was evaluated to achieve more insights into neuroprotection induced by CM following ischemic stroke. Our result demonstrated that CM3 treatment could significantly upregulate the expression of neurotrophic factors in the ischemic rat hippocampus. Neurotrophic factors are critical components of MSCs‐CM, and their role is an essential aspect of cell‐based therapy. Many studies have shown that neurotrophic factors are involved to repair of infarcted tissue by their roles in modulation of neuronal growth and survival. The neurotrophins including NGF, BDNF, and NT‐3 through binding to their Trk receptors activate PLC‐γ1 and stimulate PKC‐mediated signaling pathways, resulting in increased neuronal and synaptic plasticity.^67^ Trk receptors also by the ERK1/2 MAPK pathway activate a range of transcription factors including CREB. We previously demonstrated that hESC‐MSC‐CM treatment could elevate CREB phosphorylation in MCAO rats.^19^ The CREB activation results in overexpression of neurotrophic factors genes such as BDNF that is essential for neurogenesis and motor recovery.^68^ Interestingly, phospho‐CREB is also involved in the upregulation of anti‐apoptotic proteins Bcl2 and Bcl‐xL, which trigger neuronal survival following cerebral ischemia.^69^ Additionally, the PI3K/AKT signaling is activated by Trk receptors, which regulate cell growth, proliferation, survival, and axonal sprouting.^67^, ^70^ GDNF through interaction with its receptor, GFRα1, promotes neurogenesis by increasing the neurons number. It also involves in the survival of mature neurons.^71^

In this study, MCAO induction had no significant effect on the expression of neurogenesis and angiogenesis markers as well as neurotrophic factors in the hippocampus relative to the sham group. MCAO induction leads to territorial infarctions involving mainly striatum, frontoparietal, and temporal cortices, while may small portion of the occipital cortex, thalamus, and hypothalamus.^72^ In the present study, it seems that the hippocampus was less affected than areas such as the cortex and striatum. Therefore, the nonsignificant result of these markers in the hippocampus in response to ischemia induction is not surprising. Similarly, Nestin, DCX, BDNF, and TrkB receptor expression at mRNA and protein level on days 3, 7, and 14 after ischemia in hippocampus rats remained unchanged compared with the sham group.^73^ In addition, the sensitivity of various brain areas to ischemic insult differs based on collateral circulation, rodent strains, and ischemic induction model, so that duration of occlusion and physical features of filament affect the volume of infarction and its spread to other areas of the brain in animal models of ischemic stroke.^74^

A limitation of this study is that evaluations were conducted in young rats. One of the major problems in experimental stroke research is that many preclinical models evaluate only young male animals without any comorbidity, while aging is the most important non‐modifiable risk factor for stroke and stroke particularly affects elderly population who have various cerebrovascular risk factors.^75^

## 5 CONCLUSION

Taken together, our result suggests that repeated injections of hESC‐MSC‐CM could promote hippocampal neurogenesis and angiogenesis concomitant with inhibition of inflammation and apoptosis in ischemic brains, which may be partly mediated through neurotrophic factors expression. Further studies to elaborate the accurate efficient compounds in hESC‐MSC‐CM, underlying mechanisms of therapeutic effects, downstream signaling pathways activated by CM, and exploring its challenges would be required to propose its safety and efficacy.

## CONFLICTS OF INTEREST

None.

## Data Availability

The datasets used and/or analyzed during the current study are available from the corresponding author on reasonable request.
